# The dark side of university student entrepreneurship: Exploration of Chinese universities

**DOI:** 10.3389/fpsyg.2022.942293

**Published:** 2022-09-23

**Authors:** Xuyan Wang, Fan Chen, Hao Ni

**Affiliations:** ^1^Jing Hengyi School of Education, Hangzhou Normal University, Hangzhou, Zhejiang, China; ^2^Office of Development and Planning, Hangzhou Normal University, Hangzhou, Zhejiang, China; ^3^Center of International and Comparative Education, College of Education, Zhejiang University, Hangzhou, Zhejiang, China; ^4^China Academy of West Region Development, Zhejiang University, Hangzhou, Zhejiang, China

**Keywords:** dark side, student entrepreneurship, entrepreneurship competition, Chinese university, academic conflict

## Abstract

The dark side of entrepreneurship, especially the dark side of student entrepreneurship, has received little attention from academia. This study tries to examine the dark side of entrepreneurship among students in Chinese universities. Employing qualitative method by conducting semi-structured interview with students at the universities. Our study identifies the unproductive and destructive factors that drive the dark side of student entrepreneurship. Entrepreneurship costs that accompany students in the process of entrepreneurship are usually time pressure, academic conflict, and even health damage. There is a huge discrepancy between the knowledge given to students by universities and the knowledge required for entrepreneurship, and college students frequently lack entrepreneurial knowledge and business logic. In China, the use of inappropriate policy tools has decoupled the student entrepreneurship policies it pursues from the purpose the policies are supposed to serve. In so doing, this study contributes to the micro-level of the notion of the dark side of student entrepreneurship.

## Introduction

A broad consensus has been formed among the government, universities, and research scholars that entrepreneurship is essential for promoting economic development, science and technology, and social progress (Acs and Audretsch, 1990; Naude, 2010; Wright and Zahra, 2011; Zahra et al., 2011; Wang et al., 2021). Entrepreneurship has become an emerging research field that has gotten more scholarly attention in recent decades (Busenitz et al., 2003; Bruton et al., 2008; Welter and Lasch, 2008; Zhai et al., 2014). Popular media, as well as professional business and scientific journals, frequently mention examples of especially successful entrepreneurs and portray them as role models, which may lead to a positive bias against entrepreneurship among the general public (Beaver and Jennings, 2005; Ziemianski and Golik, 2020). In entrepreneurship research and education, this pervasive positive bias against entrepreneurship, which often stresses the benefits of becoming an entrepreneur while paying considerably less attention to the potential obstacles of establishing a business (Ziemianski and Golik, 2020). As well,

supporting successful entrepreneurial behavior is always the policy orientation, rather than assisting to repair the failure of college student entrepreneurship. Compared with the study of entrepreneurial failure, more essays focus on successful entrepreneurs examining their personality characteristics, competence structure, predictors and influence mechanism of intention and beneficial experience.

Entrepreneurship can be productive, unproductive and destructive (Baumol, 1990). Bringing attention to the dark side of entrepreneurship might be the key to promoting its flourishing (Ziemianski and Golik, 2020). Wright and Zahra proposed to conduct systematic research on entrepreneurial costs and incorporate them into the policy evaluation of entrepreneurial activities (Wright and Zahra, 2011). However, failure bias and success bias predominantly exist and characterize entrepreneurship research and its policymaking setting (Hsu et al., 2017). Fewer studies have explored the dark side of entrepreneurship (Haynes et al., 2015), let alone the dark side of student entrepreneurship. Few people talks about entrepreneurial failure, repair after failure, follow-up study, employment or re-entrepreneurship of college students. In fact, the study on the dark side of entrepreneurship education can help clarify the relationship between entrepreneurial failure and re-entrepreneurial activities of college students, and provide empirical basis for improving the policy system for entrepreneurship, the process of entrepreneurship education and the allocation of entrepreneurial resources (Zhang et al., 2021). Dimensions of dark side of entrepreneurship need to be explored (Montiel and Clark, 2018; Shepherd, 2019) and should be incorporated into entrepreneurship education (Pawel and Jakub, 2020).

To fill the research gap, the following questions will be investigated: (1) In a general sense, what are the negative effects of entrepreneurship on individual students in universities? (2) College entrepreneurs vary from traditional entrepreneurs in society since they are in the process of thinking and cognitive development as well as being tasked with knowledge acquisition. So another question that frames this research is: what are the dark sides of the phenomenon of entrepreneurship among university students in an educational sense?

## Dark side of entrepreneurship

Sociology and organizational behavior are the roots of dark side study (Vaughan, 1999; Griffin and O’Leary-Kelly, 2004; Haynes et al., 2015). Darkness has long been used as a metaphor for the human condition, its levels of awareness, its most primal instincts, and the line between life and death (Linstead et al., 2014). In the realm of organization and management studies, the dark side metaphor has been used to suggest a new concern with issues that have historically been disregarded, ignored, or concealed (Linstead et al., 2014). Their research focuses on behavior that are damaging to both humans (Andersson and Pearson, 1999; Tepper et al., 2007; Bjørkelo, 2013) and organization (Bennett and Robinson, 2000; Anand et al., 2005). In the field of organizational sociology, Vaughan (1999) believes that the dark side may be regarded as “routine non-conformity,” which can be utilized to demonstrate why things will constantly go wrong, and that dark side research can enhance policy and practice by uncovering causal structures and processes. Griffin and O’Leary-Kelly (2004) consider dark side behavior as motivated activity by an employee that has bad implications for an individual, another group of persons inside the business, or the organization itself.

A recent shift identified by Linstead et al. (2014) is that the source of darkness is within the company rather than outside. Several studies in the field of entrepreneurship research have looked at the detrimental effects of entrepreneurial personal traits on firm development. Haynes et al. (2015) has examined the potential dark side of entrepreneurs, notably the potential for and consequences of displaying greed and arrogance in various business environments. Greed and arrogance, two management characteristics associated with the “dark side” of entrepreneurial leadership, can have a negative impact on venture performance. Entrepreneurs may also have quirks such as a strong need for control over others and a tendency to be suspicious. These traits may explain their strong work ethic and high energy levels, but they may also lead to a poor management style, resulting in low employee satisfaction and decreased firm productivity (Kets de Vries, 1985). Of course, studies on entrepreneurial characteristics extend beyond the dark side to the bright side. According to Resick et al.’s (2009) research, CEO bright-side personality qualities (core self-evaluations) are favorably connected to transformational leadership, but dark-side personality characteristics (narcissism) are adversely related to contingent reward leadership. Also the dark side of social entrepreneurship is examined by Craig et al. (2019), they summarized four thematic areas according to anti- and pro- social and economic impacts of enterprise.

From a micro level, the negative psychological and emotional effects of entrepreneurial action are referred to as the dark side of entrepreneurship. Oscar et al. (2020) developed a conceptual mode of two dimensions, entrepreneur (personal) and context (cultural) to map the elements of dark entrepreneurship. Entrepreneurs may benefit from research on the dark side of entrepreneurship by reducing unpleasant feelings like anxiety and loneliness, as well as finding methods to alleviate this suffering (Shepherd, 2019). In addition to the negative effects on the entrepreneurs themselves, the impact of entrepreneurship on other members of society cannot be ignored. Entrepreneurs’ entrepreneurial behavior may cause family tension and disharmony (Beaver and Jennings, 2005). Entrepreneurship is the driving factor behind “creative destruction” in the marketplace. In this market, the entrepreneur is the “economic development leader” and innovator who can “reorganize the factors of production” and whose mission is to disrupt the market’s equilibrium creatively (Schumpeter, 1934/1990). However, the “creative destruction” of entrepreneurs can significantly affect the evolution of morality (Etzioni, 1987; Markman et al., 2016), which may lead entrepreneurs to experience moral and ethical dilemmas. The destructive nature of entrepreneurship also causes entrepreneurial behavior to be detrimental to resources owned or acquired by others, thus negatively impacting members of society (Baumol, 1996; Shepherd, 2019). Entrepreneurs’ proclivity to act as rule-breakers who push institutional limits, which has been empirically investigated as the ‘dark side’ of entrepreneurship by Zhang and Arvey (2009).

## Student entrepreneurship

In China and many other courtiers, student entrepreneurship is seen as a solution to the employment difficulties. Governments and higher education institutes tend to promote employment rate by guiding college students to start a business. While, relevant reports show that the success rate of Chinese college students in starting businesses is only about 5%, less than a quarter of that of college students of the USA (Li et al., 2019). In addition to receiving applause and money, college students face unusual pressure when establishing start-ups. Kai-Fu Lee, a role model for college students entrepreneurs in China, also admitted that college students in China are under greater pressure to start their own businesses than in the United States. Most Chinese students will get negative comments if they tell their relatives and friends they want to start a business. Especially they will suffer from family’s stress. They would rather let them find a so-called “good job,” like being a teacher or civil servant.

According to the 2021 China College Student Entrepreneurship Report, in the survey to 10,791 students, 96.1% have had the idea and intention to start a business, and 14% have started a business or are preparing to do so (He, 2022). Even with an advantageous business climate, college students tend to fail more than successful entrepreneurship. for various reasons, many college students can’t get out of the dark side, but fall into various problems. Firstly, high risk and high failure rate make the college student entrepreneurs frequently encounter various economic and physical and mental setbacks and other pressure scenarios, which have a negative impact on the cognition of the entrepreneurs, forcing the entrepreneurs to suddenly retreat from fear, and further trigger the withdrawal of entrepreneurship. According to Cao’s (2021) survey to 290 samples, College students’ entrepreneurial pressure has a positive impact on their entrepreneurial withdrawal willingness, and entrepreneurial self-efficacy plays a mediating role between them two. The entrepreneurial pressure perceived by college students mainly comes from entrepreneurial involvement, knowledge reserve, competitive intensity, management responsibility and resource demand. Besides, family support regulates the mediated relation between entrepreneurial stress and entrepreneurial withdrawal willingness.

For students and Higher Education Institutions (HEIs), another dilemma is to balance the entrepreneurial activities and academic performance, which already were proved to be mutually influenced (Gralewski and Karwowski, 2012). It is recognized as emphasizing the advantages of becoming an entrepreneur and giving considerably less attention to potential downsides (Pawel and Jakub, 2020). Bandera et al. (2021) explores what dangers might entrepreneurship education pose to the school and to students themselves. Among those, one danger and worry is that schools become entrepreneurial institutions in a way that is not aligned with their mission.

## Entrepreneurship education in China

The Chinese government began to develop entrepreneurial education in 1998. Following that, the State Council, the Ministry of Education, and other government ministries launched a slew of policies, rules, and regulations aimed at encouraging entrepreneurship and the ecological growth of entrepreneurship education (Xu and Wang, 2016). These policies have had a significant impact on the development of national-wide entrepreneurship education throughout China, see Table 1.

**TABLE 1:** Milestones of entrepreneurship education policies and initiatives in China.

Year	Policies and initiatives	Goals to be achieved
1998	Action Plan for the Revitalization of Education for the 21st Century	Developing entrepreneurship education among faculty and students
2002	Take Tsinghua University, China’s Renmin University, and nine other institutions as examples of entrepreneurial education pilots	Kicked off the research and practice of entrepreneurship education in Chinese universities
2005	KAB Entrepreneurship Education Program (China) established	Cooperation with international organizations to serve Chinese youth entrepreneurship and employment
2010	College Students’ Entrepreneurship leading plan	Popularize entrepreneurship education, strengthen entrepreneurship training, and provide support and services for college students to start their own businesses
2010	Opinions of the Ministry of Education on vigorously promoting innovation and entrepreneurship education in higher education and independent entrepreneurship among college students	Promote innovation and entrepreneurship education in higher education, strengthen innovation and entrepreneurship education curriculum system and faculty construction, and widely carry out innovation and entrepreneurship practice activities
2012	Basic Requirements for Teaching Entrepreneurship Education in General Undergraduate Schools (for Trial Implementation)	Universities are required to offer mandatory entrepreneurship courses for all students
2015	Implementation Opinions of the General Office of the State Council on Deepening the Reform of Innovation and Entrepreneurship Education in Higher Education	Vigorously promote entrepreneurship education and strengthen the government’s macro guidance on entrepreneurship education
2021	Guidelines of The General Office of the State Council on Further supporting College Students’ innovation and Entrepreneurship	Support college students in enhancing their ability to innovate and start businesses, and support college graduates in starting businesses and finding jobs

These entrepreneurial policies and initiatives launched by the Chinese government have achieved a lot of attention and positive feedback. Chinese universities have established entrepreneurship education management organizations, conducted entrepreneurship teacher training, offered entrepreneurship courses to students, and launched practical entrepreneurship activities for college students as a result of China’s entrepreneurship education policy (Dong, 2012).

Entrepreneurship competition, in addition to entrepreneurship classes, are an inseparable topic for Chinese students interested in starting their own firm. Up to now, China has formed a complex system of entrepreneurship competitions, with different categories of innovation and entrepreneurship competitions set up from national to provincial, municipal, and university levels (see Table 2).

**TABLE 2:** Various types of innovation and entrepreneurship competition projects at all levels (selected).

National level	◆ China Innovation and Entrepreneurship Competition; ◆ “Creating Youth” National College Student Entrepreneurship Competition; ◆ College Student Innovation and Entrepreneurship Competition; ◆ China International “Internet+” College Student Innovation and Entrepreneurship Competition; ◆ National College Business Elite Challenge Exhibition Professional Innovation and Entrepreneurship Practice Competition; ◆ China College Student Public Relations Planning and Entrepreneurship Competition.
Provincial level (Zhejiang Province)	◆ Zhejiang Province University Student Science and Technology Innovation Activity Plan Competition; ◆ Zhejiang Province “Challenge Cup” Student Entrepreneurship Plan Competition; ◆ Zhejiang Province “Internet+” Student Innovation and Entrepreneurship Competition.
Municipal level (Hangzhou)	◆ Hangzhou Student Entrepreneurship Competition; ◆ Hangzhou Innovation and Entrepreneurship Competition.
Campus level (AU)	◆ AU “Dandelion” Student Entrepreneurship Competition; ◆ AU “Challenge Cup” Student Entrepreneurship Plan Competition; ◆ AU Student Career Planning and Entrepreneurship Competition.
Campus level (DU)	◆ Starlight Award; ◆ Undergraduate Innovation Ability Enhancement Project; ◆ DU “Hope Cup” Student Entrepreneurship Plan Competition.

## Research design and methods

To answer the questions posed by the study, we employed qualitative research methods. When compared to quantitative research, qualitative research may go deeper into participants’ inner experiences, capture subtleties, and complexity that quantitative approaches can’t, and reveal and explain phenomena that aren’t well understood on the surface. There are few studies on the negative aspects of college student entrepreneurship, thus qualitative research methodologies are a good way to start (Corbin and Strauss, 1996). Semi-structured interviews, participatory observation, and document analysis are among the data collection methodologies used in this study (Carvalho et al., 2010; Wang et al., 2021). Interviews’ interactive aspect helps researchers to analyze complicated subjects that would be difficult to investigate in depth with questionnaires (Wang et al., 2021). We conducted in-depth interviews with 15 entrepreneurial college and graduate students using a grounded theory approach and coded the interviews for analysis (Charmaz, 2006). To ensure the quality and scientific validity of the study, a variety of sampling methods were used to determine the site and respondents in this study.

## Site selection and access

In selecting the study sites, this study adopted a typical case sampling method. We selected Zhejiang Province as the study site under investigation, which is considered as China’s Silicon Valley and has giant Chinese Internet companies such as Alibaba Group and NetEase. In 2021, three Chinese unicorns are listed in the top 10 of the global unicorn list, two of which are in Hangzhou, Zhejiang Province, namely, Ant Group and Cainiao Network. Zhejiang Province is a strong province in terms of scale of higher education, and she also implements the strategy of “Rejuvenating Zhejiang *via* Science and Education,” to pursue sustainable development. Thus, in 2021, there are 129,860 graduate students (including part-time), including 19,447 doctoral and 110,413 master’s students; there are 1,210,300 undergraduate students, including 683,700 university students and 526,600 higher vocational students (Department of Education of Zhejiang Province, 2022). In addition, Zhejiang Province is a leader in EE practices at the national university level by building a series of EE programs, such as building an innovation and entrepreneurship talent training system, promoting the construction of innovation and entrepreneurship curriculum system and teaching reform, strengthening innovation and entrepreneurship guidance services for college students, and improving the financial support system and policy guarantee system for innovation and entrepreneurship (Mei and Symaco, 2022).

In selecting the universities in Zhejiang Province, we adopted a stratified purposive sampling method. We selected four universities in Zhejiang Province with different levels, nature, scale, and characteristics of operation as the target universities for our sampling, and find student entrepreneurs from these universities. AU is one of China’s top comprehensive universities, dubbed the “Stanford of the East,” and has been chosen for numerous national initiatives in China, including the “Double First Class,” “985,” and “211” programs. The level of AU’s research, globalization, and alumni network are all world-class. BU is one of the best art colleges in China, and its artistic achievements in music, painting and design are second to none in China. CU is a comprehensive university with a strong focus on science and engineering disciplines and is the second-best comprehensive university in Zhejiang Province after AU. DU is a comprehensive university with a strong focus on the humanities. Its overall research strength is not as strong as CU’s, and it is ranked around 100th in China’s university rankings, but the university is very distinctive in the cultivation and practice of innovative and entrepreneurial talents and has produced outstanding entrepreneurs such as Jack Ma.

## Data collection strategies and sampling

When searching for student entrepreneurs in the target universities, we adopt a maximum variance sampling method. As shown in Table 3, the sampling criteria of this study considered and covered factors such as gender, academic background, grade, major, field of entrepreneurship, current status of entrepreneurship, success or failure of entrepreneurship, and number of times of entrepreneurship of the respondents. The selected respondents were from different universities, with one-third of them graduate students and the others undergraduates. Among them, 40% are female. By comparing their major and entrepreneurial field, we can find that 6 interviewees are in the same or quite close field. In total, 60% of them didn’t choose to start a business in their field of expertise. More importantly, 5 of them are serial entrepreneurs who have started a business for 2 to 4 times. Among them, 2 undergraduates have started businesses for 4 times and are both male students. During the sampling process, we employed a variety of strategies to obtain respondents. To obtain interviews, the initial method was to attend entrepreneurial events or workshops at the university. The second tactic was to recruit entrepreneurial students through faculty members who were in charge of entrepreneurial activity. The third technique was to locate interviewees *via* the Internet by searching for news stories about excellent entrepreneurial students at the chosen university and contacting them. By snowballing, the fourth method was to ask interviewees to introduce us to interviewers they knew. All participants were subjected to semi-structured interviews performed by the first author. At the completion of each sample, we analyzed the content of the interviews, and based on the results of the analysis we searched for the next interviewee who would fit the theoretical sample. Sampling for this study was stopped when the data reached theoretical saturation and a total of 15 samples were obtained for this study. COVID-19 influenced four of the 15 participants to be questioned over the phone, while the remaining 11 were interviewed in person. The goal of our study was explained to all respondents, and their personal information, such as their school and startup firm, was anonymized at their request. Each interview ranged from 30 to 90 min, and all of them were recorded and preserved electronically. The information of the respondents is shown in Table 3. All the participants are interviewed in spring of 2022.

**TABLE 3:** Code list of interviewees.

**Code**	**Gender**	**Identity**	**Year**	**Major**	**Entrepreneurship projects**	**Serial entrepreneur**	**Startup status**
S1	Female	Undergraduate	Junior	Biosystems Engineering	Internet of things	N.A.	Quit
S2	Female	Post-graduate	Master student	Architecture	Study abroad services	N.A.	Ongoing
S3	Female	Undergraduate	Senior	English	Education services	N.A.	Ongoing
S4	Male	Post-graduate	Doctor student	Geology	Costume rental	N.A.	Quit
S5	Female	Undergraduate	Junior	Education	Public sector entrepreneurship: museum education	N.A.	Ongoing
S6	Female	Undergraduate	Sophomore	E-commerce	Public sector entrepreneurship: rural education	N.A.	Ongoing
S7	Female	Undergraduate	Sophomore	Education	Social entrepreneurship: rural education	N.A.	Ongoing
S8	Male	Undergraduate	Sophomore	E-commerce	Business incubator	2	Ongoing
S9	Male	Undergraduate	Sophomore	E-commerce	UAV education and training	N.A.	Ongoing
S10	Male	Undergraduate	Junior	Clinical Medicine	Biotechnology	N.A.	Ongoing
S11	Male	Post-graduate	Doctor student	Communication and Media	Biotechnology	N.A.	Ongoing
S12	Male	Undergraduate	Graduated for 5 years	Big Data Analytics	Visual design	4	Ongoing
S13	Male	Undergraduate	Junior-Delayed graduation for 2 years	Nursing	HRO Process Outsourcing	4	Ongoing
S14	Male	Post-graduate	Doctor student	Painting	Household Products	2	Ongoing
S15	Male	Post-graduate	Doctor- Graduated for 2 years	Artistic Theory	Media	3	Quit

The number in the “serial entrepreneurs” column indicates the number of times the entrepreneur has started a business.

## Findings

### The negative side of entrepreneurship experienced by individual students

#### The conflict between entrepreneurship and studies

Entrepreneurship has a direct influence on students by squeezing out their time and energy, and placing pressure on their schoolwork. Students’ time and energy devoted to entrepreneurship can directly contribute to poor academic performance and even cause absenteeism, failure, and suspension, especially for junior undergraduates who have heavy study burdens. When discussing his third venture, S15, a serial entrepreneur who had three failed companies as a student, highlighted the time pressure they faced as follows.

*I achieved a personal record by sleeping two hours a day for 15 days straight in order to edit videos overnight. We were on the verge of taking the firm to the next stage when we saw a surge in sales.*

When students are confronted with an academic conflict, they employ a range of tactics to alleviate the conflict and the stress it causes, even if these strategies are not conducive to their growth. For example, S9, an undergraduate entrepreneur, explained how he frequently missed courses and had considered giving up his business.

*Last semester I really crashed all over the place, my time was jammed with companies, classes and startup competitions. Last semester, I was all over the place, with companies, schools, and startup competition cramming my schedule. Due to the time constraint, I had to drop several classes in order to perform various off-campus activities. I missed two lessons last Monday to attend a meeting at Sunshine Race. Another thing is energy, you will be sidetracked when doing homework, and there will be some things that will take up your homework time. Last but not least, I failed one course last semester, which has had an impact on my marks.*

Other self-adjustment tactics mentioned by the respondents included S1 abandoning entrepreneurship, S8 taking final examinations in the form of the final blitz, and S6 and S7 both staying up late or giving up their leisure time. S13 highlighted his attempt to switch majors. However, his major was unable to change due to his poor grades. He then chose to take a break from his studies to ease his academic conflicts. He has so far finished 6 years of undergraduate study, which should have taken him 4 years, and he is still on leave.

The negative mental and physical effects of stress on students. When faced with the stress of starting a business they can’t handle, students commonly suffer a number of negative emotions such as breakdown, depression, irritation, anxiety, and self-doubt. S6, a female undergraduate entrepreneur, gave us this description of the negative emotions she experienced before the interview:

*It’s vexing, very annoying. I’ve been crying a lot lately, and I’m terribly sad. But I don’t generally weep right away when this happens. This item will build up in my heart for a while before exploding (cry).*

Students’ health is affected by stress and bad emotions, which is mirrored in their physical functioning, with many of the students interviewed admitting to being tired. S6 continued by saying that:

*I had a lot of physical changes, my skin would get bad, I had dark circles under my eyes, and I had acne, my whole body was emaciated, and I would even have stomach pains.*

S13, an undergraduate serial entrepreneur who started his business in his senior year of high school, described his physical changes in this way.

*I’m really stressed, and I can say I’m a little deformed (physically fat). Mentally it’s probably deformed as well. Yes, it feels like I’m not quite sure I want to do this, so the easiest thing to do is to have self-doubt. I keep asking myself if I want to do this. Physically, I’ve gained weight, which is stress-related obesity, and this stress is related to entrepreneurial stress as well as academic stress. Finally, I had facial palsy, which is neurological facial palsy caused by psychological and mental stress. Later, in order to treat facial palsy, the medicine I took had side effects, and my body became even fatter.*

### Misalignment of student knowledge with what is needed for entrepreneurship

#### Lack of entrepreneurial knowledge and business logic

Universities are actively promoting entrepreneurship education and arranging activities such as entrepreneurship competitions to boost student entrepreneurship, according to the findings of the study. However, these initiatives merely ignite students’ enthusiasm for entrepreneurship, not equipping them with the knowledge and competencies required to launch their own businesses. In the first statement of his interview, S15, a serial entrepreneur who had three failed business ventures as a student and is now employed at a university, emphasized this point:

*Entrepreneurship is a matter of dealing with business and society. And anything you learn in the course of school has nothing to do with these two things. So entrepreneurship is a distinct area of knowledge. I’m 34 years old and haven’t dealt with society much until I started working. I realized that what students study has nothing to do with business. What a student learns is a blind spot for entrepreneurship.*

Of course, S15’s enterprise failed, but the viewpoints of successful entrepreneurial students back up this assertion. For example, S13, an undergraduate serial entrepreneur who began his business in his third year of high school, when talking about the positive impact of entrepreneurship on him, he noted that:

*I have learned many things in the business world, including communication with customers, employee management, and legal arbitration. I have dealt with all kinds of disputes, including financial and taxation problems. I have also experienced competition from competitors, the maintenance of the relationship between the A-side and the customer, and various work-related injuries and accidents. You will never learn these if you do not have experience, and this is a pretty systematic thing.*

S11, a successful doctoral candidate entrepreneur with a company turnover of 200 million RMB by 2021, who has worked as an executive in a public company for 3 years, and considers himself not a traditional student entrepreneur, expresses his views on student entrepreneurship by revealing the essence of entrepreneurship:

*Can students start their own businesses? Entrepreneurship is the type of thing that requires a high level of expertise and resources. You have to have the skills, the team, and the resources. You also have to get whatever supply chain open, you have to have that channel resources, and those are not things that students can handle. If the student’s family or mentor has those resources, it’s a different story. I am not a student in the traditional sense, prior to beginning my Ph.D. programme, I handled several hundred million yuan in assets for a public corporation. My current company’s CFO was poached from that firm. Student entrepreneurship is actually rather challenging, and if you lack these resources, your chances of success are slim.*

It is true that the lack of knowledge and resources for effective entrepreneurship lays the groundwork for students’ entrepreneurial failures. For example, S15, for his part, had two failed entrepreneurial experiences caused by a lack of legal awareness and knowledge. He gives us this warning through his own painful lessons.

*Based on my experience, I believe it is critical to first popularize the basic laws for students. My initial business was on Taobao, however, our online store was closed because of malicious ‘shuadan’ (a method of obtaining positive evaluations that Taobao does not allow). My partner remains on Taobao’s permanent blacklist to this day. He will never be allowed to start another store on Taobao. My second business venture was rather miserable, as I was working on a capsule hostel, but Chinese law was unclear about this business so far. China’s fire laws are particularly strict, stipulating that a maximum of five people can live in a 40-square-foot house, and if six people live there, it is illegal to rent in a group. But the reason for the existence of this kind of hostel is the high density and high convenience, and this model itself is in conflict with the Chinese laws and regulations. We didn’t know that at the time. We thought it would be fine as long as we could ensure the safety of the tenants and pay attention to fire prevention every day. Our three partners invested more than 200,000 yuan, but in the end, we lost all our money. Some business coaching also mentioned that the law is important, but we grew up under the care of our parents when we were young, any lecture is useless, you have to let them know about these live cases.*

Similarly there are many other students who have faced failure or difficulties due to lack of entrepreneurial knowledge and business logic. For example, S4’s lack of awareness about the entrepreneurial team’s benefit allocation at the start of his participation in the venture led to issues he couldn’t deal with and a subsequent withdrawal from the business. S14 is a serial entrepreneur who is also a Ph.D student. When talking about his first entrepreneurial experience, he flirted with the idea of his company being a product development department. He attributed the company’s failure to their team’s lack of knowledge of business operations and their company’s lack of basic structure. Indeed, the ability to develop products is an advantage of students over traditional entrepreneurs, but it may also restrict students’ entrepreneurial ideas. S10, a successful biotechnology undergraduate entrepreneur who was awarded the Star of Innovation and Entrepreneurship in Zhejiang Province and won a silver medal in a national entrepreneurship competition, showed us the transformation of his ideas after starting his own business.

*My thinking has changed drastically, I used to be very R&D oriented. But now my scientific thinking has changed, my customer’s needs are the first, that is, what the customer needs, I provide him with what kind of products.*

#### Students’ entrepreneurial needs are not met by entrepreneurship courses

Almost every respondent expressed their disapproval of the quality of entrepreneurship courses currently offered in universities. Students would rate entrepreneurship courses in terms of “*shui ke.” Shui ke* refers to a phenomenon that happens in Chinese university curricula. Typical characteristics of such courses include dull course content, sloppy course management, high course performance points, etc. Students receive little from such courses, and they are not helpful to their professional development. Entrepreneurship courses are usually general education courses at universities. Due to the lack of faculty, entrepreneurship courses are usually conducted in the form of online courses. Such online courses lack effective course management, and the actual effectiveness of the courses is a concern. As S5, a female social entrepreneur, described the entrepreneurship course this way:

*‘Entrepreneurial practice’ is an online course anyway, it is actually very watery. We just need to fill up his credits online and finish the class on the line (here means play all the videos of the course). There is no exam in this class. The course work is a few questions after each small video, these questions have three options, just choose any one of them. Yes, the quality of the course is not enough.*

S7, another social entrepreneur, explains the students’ attitude toward the entrepreneurship course from a different angle.

*I think his class (Entrepreneurship Fundamentals course) is pretty good. Because this course is an online course, I did not listen to much, anyway, his final assessment method is also very simple, I can easily get a score of 4.5. We felt that entrepreneurship was far away from us, and we didn’t take the course seriously because we didn’t think it would help us in our careers when we took it. Everyone is ‘shua ke’, that is, playing the videos of this course on their phones while they are in other classes.*

The entrepreneurship courses mentioned by the two students above are only general education entrepreneurship courses offered by universities for the average student, and at best, they can only serve as an initiation to entrepreneurship. For real entrepreneurs, even the courses offered by business schools cannot meet their entrepreneurial needs. For example, in S8, a student entrepreneur in E-commerce offered that:

*For me, academics is just a performance point, the books are no longer enough to satisfy me. For example, we have a class called e-commerce website building. There’s a class called that e-commerce website building. This course will only give you the basic operation process, but actually when you practice it is completely different. I have to put it into a whole project, schedule it, and interface with people from all sides. There is a big difference between the book and the practical needs of entrepreneurship.*

S5 mentions the important point that even if universities have a well-developed business curriculum and students are able to learn sets of business theories, entrepreneurs will still face a gap between theoretical business knowledge and practical entrepreneurship. S13, a serial entrepreneur, reinforces this point:

*What is taught in college is a completely different set than what is taught in the real world. What is taught in college is right. Those business theories are the golden fruits of a thousand refinements. But this thing is useless, it cannot guide the practice, especially the practice of small businesses. For example, the market research in marketing may be public companies can follow his grand system to develop. Like Huawei, which has its own research institute, can consider these issues, that is, listed away all the customers, split the market demand, then break through the market demand one by one. These are fairly theoretical concepts that are difficult to apply in practice. Of course, these theories can be applied, but doing so will reduce your efficiency.*

Furthermore, universities lack an established entrepreneurial ecosystem, and students lack adequate expert assistance for their extracurricular entrepreneurial endeavors. On the one hand, this is due to the nature of venture capital in China, where many venture capitalists are unwilling to participate in startups or will only engage in and mentor students at top universities, leaving the majority of students without access to truly helpful entrepreneurial coaching. Students, on the other hand, typically only have access to entrepreneurs or other professionals through entrepreneurship competitions, making expert coaching in extracurricular activities unproductive.

### Decoupling of educational policies from its purposes

Decoupling is a phenomenon in which an organization minimizes controversy and conflict by disconnecting from institutional structures with the aim of gaining legitimacy and maintaining standardization and formal structures (Meyer and Rowan, 1977). Organizations frequently use decoupling to manage with stress and test out new strategies (Oliver, 1991; Boxenbaum and Jonsson, 2017). Entrepreneurship competitions are now the most popular way for Chinese university students to get involved in entrepreneurial activities. In order to be able to better promote student entrepreneurship, China has developed a system of entrepreneurship competitions of various levels and types. These are well-intentioned measures. The use of project award as a measure of the effectiveness of entrepreneurial activities carried out in schools, on the other hand, decouples the student entrepreneurship policy from its intended goals. All of the students interviewed have competed in business competitions. Almost all respondents, with the exception of S10, had a negative view of entrepreneurial competitions. Most students use entrepreneurship competitions as a way to enhance their academic GPA, boost their resume, earn scholarships, and get qualify for graduate school without taking the graduate entrance examination, according to these students. In addition, the multiple forms of entrepreneurial competitions consume a lot of time and energy, and many students participate in multiple entrepreneurial competitions at the same time. Students also usually take the same entrepreneurial project to other non-entrepreneurial projects, such as innovation competitions and career planning competitions. Neither the organizers nor the students involved appear to be concerned about the entrepreneurial project’s success. S12, a serial entrepreneur and manager of a business incubator, reveals the true state of some startup competitions.

*Some students are just too busy to play the competition, he actually did not think how to land the project. competition is actually a performance, those projects are bragging to see who speaks beautifully and portray a bright future. great people are not willing to be judges, they think it is a waste of time.*

The conditions for success in entrepreneurship competitions were highlighted multiple times by students. They usually involve the elegance of the competition PowerPoint, the number of patents, papers and celebrities endorsed behind the proposal, the efficacy of the student’s prowess during the pitch, etc, while projects that are truly genuinely entrepreneurial do not earn the judges’ favor. So much so that the first author overheard students flirting with the entrepreneurship competition as a PowerPoint competition during interviews. These entrepreneurial competitions are not well received by real student entrepreneurs. They compete in these tournaments because their lecturers have invited them to win award and honors for the university. S11, a Ph.D. entrepreneur whose company grossed $200 million in 2021, for example, demonstrates the fact that:

*I was invited to the entrepreneurship competition by the Institute of Innovation and Entrepreneurship. Everyone is basically around the Challenge Cup and the Internet* + *competitions. Teachers will come to you and tell you to go to the competition if the school has a good student entrepreneurship project. I have a good relationship with the dean of the School of Innovation and Entrepreneurship, he came to me and I did him a favor. Participating in this competition is not of any use to me personally. I am not responsible for all the preparation of materials in the competition, I will just show up during the pitch.*

The research also discovered an intriguing phenomenon. Some university-based entrepreneurial programs have a long history. Some of the so-called “entrepreneurship” programs resemble student groups more than anything else. These programs are usually faculty-led and recruit new members each year, mainly freshmen, with just a small number of students sticking on for their second year and becoming the program’s “leader.” These new project leaders then recruit and mentor new students in order to develop them as project leaders. These enterprises are frequently passed down from generation to generation, and one of the longest entrepreneurial projects we met with at the university has been in existence for 13 years.

*This entrepreneurship program can be passed on to underclassmen. Some national entrepreneurship programs require freshmen to be part of the program. So when I was a freshman, I had seniors come to me to participate in the national entrepreneurship program, which aims to ensure the legacy of the program.*

Such programs are frequently structured to serve as competition award, and some teachers with extensive experience coaching entrepreneurship competitions typically have a dozen or more competition projects on hand. Teachers can benefit from mentoring students to win entrepreneurship project award, such as receiving merit or promotions.

Entrepreneurship competitions aren’t wholly pointless, though. Entrepreneurship competitions have helped some students launch their entrepreneurial concepts by providing incubation and proof-of-concept opportunities. For example, S10, a silver medal winner of the *International “Internet*+*” Student Innovation and Entrepreneurship Competition*, who was named *Typical Person of Zhejiang Province Student Employment and Entrepreneurship*, caught this.

*The competition was the biggest factor that pushed me to start my own business. In the beginning, I simply entered the competition for the sake of it. Then I found that my project could really be implemented, and then I started to implement it. I think the biggest role of the competition is to practice with the competition. Without the process of the competition, which helped me polish my project step by step, I might have failed in the first step of my business, and the whole process of the competition helped me to prove and implement my business project.*

## Discussion

The study of the dark side of student entrepreneurship is more likely to identify the interpersonal and institutional dilemmas of this phenomena so that its dark side can be better transformed into the bright side. Of course, the understanding of the dark side of student entrepreneurship is not absolute, as the proposed dark side may also be the bright side. There is a dividing line between the two, but this line is blurred, and just as entrepreneurial self-confidence can become arrogance when taken to the extreme, the bright side can also turn to the dark side (Kets de Vries, 1985; Haynes et al., 2015).

Dialectics is needed when looking at the negative effects of entrepreneurship on students. These negative effects will indeed bring some dark emotional experience and physical reflection to students, but they will also bring bright side to students, such as the improvement of ability and confidence. Students can also eliminate the negative impact of entrepreneurship through various ways, such as quitting entrepreneurship, improving study and work efficiency, and dropping out of school, which gives students more knowledge and experience of learning, social and professional development.

Another finding is the serious mismatch between students’ knowledge system and the knowledge system needed for starting a business. Is student suitable for entrepreneurial activities? Entrepreneurship is a narrow escape, and successful entrepreneurs have personal connections, funds, experience and other support that ordinary wage earners do not have. Policies, courses and guidance for university students only ignited students’ entrepreneurial awareness, but they didn’t really give them a complete knowledge system of entrepreneurship. The market is real and cruel, and entrepreneurs will not be treated differently because of their student status. Due to the lack of entrepreneurial knowledge, business logic and resources, most student entrepreneurs fail and eventually bear the trial and error cost of large-scale entrepreneurial policies. Weber warned that a society dominated by legal-rational organizations would suffer undesirable effects (Weber, 1998). Any system of action inevitably generates secondary consequences that run counter to its objectives and this is the basis of the dark side that any organizational action possesses (Merton, 1968). A diverse sociological basis of organizational, public, and social control agents is required to affect policy (Vaughan, 1999). Universities act more as agents in the implementation of entrepreneurship education policies in China, but since the entire entrepreneurial ecosystem does not develop fully in universities, this undeveloped agent status has a negative impact on policy implementation. Currently, the entrepreneurship education in Chinese universities still suffer from a scarcity of entrepreneurship education instructors, a scarcity of diverse entrepreneurship education courses, and a lack of enthusiasm for entrepreneurs’ involvement (Mei and Xu, 2009; Huang and Huang, 2019). Although entrepreneurship education is currently in full swing in Chinese universities, there is a problem with emphasizing quantity over quality and scale over substance, and entrepreneurship education in universities has a highly convergent organizational structure, faculty strength, and practice platform (Xu et al., 2021). To strengthen their legitimacy and survival chances, organizations are compelled to integrate practices and procedures established by prevalent rationalized notions of organizational activity and institutionalized in society (Meyer and Rowan, 1977). One can see the efforts of Chinese universities to adapt to the national institutional environment in order to access resources. However, inconsistencies between its internal structure (entrepreneurial ecology) and work needs result in a decoupling of policy and practice, resulting in the chaos explored in the study. To be more specific, through organizing and participating the competitions, universities are trying to get more outreach opportunities and resources; teachers hope to get promoted in annual performance appraisal by mentoring; students expect to improve academic performance. The mismatching of policy goals and policy tools leads to the emergence of all kinds of chaos in college entrepreneurship and education, which in some degree explains the phenomenon of low actual operation rate of venture project in China (Huang and Huang, 2019).

## Conclusion

Focusing on the dark side of student entrepreneurship, this study has analyzed the personal impact of entrepreneurship on students and the issues that China has experienced in implementing student entrepreneurship policies. Overall, the negative effects of entrepreneurship on individual college students can be addressed through a variety of strategies. However, implementing student entrepreneurship policies on a large scale needs to be approached with caution. While this is only a Chinese experience, the Chinese education system may not be applicable to the rest of the world. One thing is certain, however, that while entrepreneurship is an emerging as well as hotly debated topic in the research field, it is dangerous to pursue student entrepreneurship policies on a large scale, regardless of location, until entrepreneurship research is mature and an entrepreneurial ecology is established. In addition, while every effort has been made to ensure the scientific validity of the findings of this study using an integrated and comprehensive sampling method, it is not possible to exhaust the dark side of entrepreneurship among Chinese university students in one study, which is a limitation of this study. Unlike quantitative research using large-scale probability sampling, qualitative research does not aim to extrapolate to the sample as a whole; it aims to gain insight into the intrinsic experiences of respondents in order to gain a more in-depth and detailed interpretive understanding (Chen, 2000), and to provide experience and evidence in areas where the research base is underdeveloped, and this is where the value of this study lies. Research on the dark side of student entrepreneurship needs to be further developed, and there remains tremendous scope and possibility for future research on the dark side of entrepreneurship.

## Data availability statement

The original contributions presented in this study are included in the article/supplementary material, further inquiries can be directed to the corresponding author.

## Author contributions

XW completed the entire study design, interview data collection, and thesis writing. HN helped to write part of the literature review and helped to revise and improve the manuscript. FC gave ideas for the thesis writing. All authors contributed to the article and approved the submitted version.
